# Supplementary material for: Modelling Skylarks (Alauda arvensis) to Predict Impacts of Changes in Land Management and Policy: Development and Testing of an Agent-Based Model
Source: PLoS One. 2013 Jun 6;8(6):e65803. doi: 10.1371/journal.pone.0065803 (PMC3675089; doi:10.1371/journal.pone.0065803)
Supplement: Supporting Information S4 — The skylark ODdox as a zipped archive. (ZIP) [file pone.0065803.s004.zip › Skylark_ODdox/tov__declaration_8h.html]

ALMaSS Skylark ODdox: tov\_declaration.h File Reference


ALMaSS Skylark ODdox
 2.0


- Main Page
- Related Pages
- Classes
- Files

- File List
- File Members

Enumerations

tov\_declaration.h File Reference

|  |  |
| --- | --- |
| Enumerations | |
| enum | TTypesOfVegetation {     tov\_Carrots = 0, tov\_CloverGrassGrazed1, tov\_CloverGrassGrazed2, tov\_FieldPeas,     tov\_FodderBeet, tov\_Maize, tov\_NaturalGrass, tov\_NoGrowth,     tov\_None, tov\_Oats, tov\_OBarleyPeaCloverGrass, tov\_OCarrots,     tov\_OCloverGrassGrazed1, tov\_OCloverGrassGrazed2, tov\_OCloverGrassSilage1, tov\_OFieldPeas,     tov\_OFirstYearDanger, tov\_OGrazingPigs, tov\_OOats, tov\_OPermanentGrassGrazed,     tov\_OPotatoes, tov\_OSeedGrass1, tov\_OSeedGrass2, tov\_OSetaside,     tov\_OSpringBarley, tov\_OSpringBarleyClover, tov\_OSpringBarleyGrass, tov\_OSpringBarleyPigs,     tov\_OTriticale, tov\_OWinterBarley, tov\_OWinterRape, tov\_OWinterRye,     tov\_OWinterWheatUndersown, tov\_PermanentGrassGrazed, tov\_PermanentGrassTussocky, tov\_PermanentSetaside,     tov\_Potatoes, tov\_PotatoesIndustry, tov\_SeedGrass1, tov\_SeedGrass2,     tov\_Setaside, tov\_SpringBarley, tov\_SpringBarleyCloverGrass, tov\_SpringBarleyGrass,     tov\_SpringBarleySeed, tov\_SpringBarleySilage, tov\_SpringRape, tov\_SpringWheat,     tov\_Triticale, tov\_WinterBarley, tov\_WinterRape, tov\_WinterRye,     tov\_WinterWheat, tov\_WWheatPControl, tov\_WWheatPToxicControl, tov\_WWheatPTreatment,     tov\_AgroChemIndustryCereal, tov\_WinterWheatShort, tov\_Orchard, tov\_OSBarleySilage,     tov\_OFieldPeasSilage, tov\_FieldPeasStrigling, tov\_MaizeStrigling, tov\_SpringBarleyStrigling,     tov\_SpringBarleyCloverGrassStrigling, tov\_WinterBarleyStrigling, tov\_WinterRapeStrigling, tov\_WinterRyeStrigling,     tov\_WinterWheatStrigling, tov\_SpringBarleyPeaCloverGrassStrigling, tov\_YoungForest, tov\_SpringBarleyStriglingSingle,     tov\_SpringBarleyStriglingCulm, tov\_WinterWheatStriglingSingle, tov\_WinterWheatStriglingCulm, tov\_MaizeSilage,     tov\_FodderGrass, tov\_Lawn, tov\_PermanentGrassLowYield, tov\_SpringBarleyPTreatment,     tov\_OSpringBarleyExt, tov\_OWinterWheatUndersownExt, tov\_OWinterBarleyExt, tov\_OMaizeSilage,     tov\_SpringBarleySKManagement, tov\_Undefined   } |

---

## Enumeration Type Documentation

enum TTypesOfVegetation

**Enumerator:**
:   |  |  |
    | --- | --- |
    | *tov\_Carrots* |  |
    | *tov\_CloverGrassGrazed1* |  |
    | *tov\_CloverGrassGrazed2* |  |
    | *tov\_FieldPeas* |  |
    | *tov\_FodderBeet* |  |
    | *tov\_Maize* |  |
    | *tov\_NaturalGrass* |  |
    | *tov\_NoGrowth* |  |
    | *tov\_None* |  |
    | *tov\_Oats* |  |
    | *tov\_OBarleyPeaCloverGrass* |  |
    | *tov\_OCarrots* |  |
    | *tov\_OCloverGrassGrazed1* |  |
    | *tov\_OCloverGrassGrazed2* |  |
    | *tov\_OCloverGrassSilage1* |  |
    | *tov\_OFieldPeas* |  |
    | *tov\_OFirstYearDanger* |  |
    | *tov\_OGrazingPigs* |  |
    | *tov\_OOats* |  |
    | *tov\_OPermanentGrassGrazed* |  |
    | *tov\_OPotatoes* |  |
    | *tov\_OSeedGrass1* |  |
    | *tov\_OSeedGrass2* |  |
    | *tov\_OSetaside* |  |
    | *tov\_OSpringBarley* |  |
    | *tov\_OSpringBarleyClover* |  |
    | *tov\_OSpringBarleyGrass* |  |
    | *tov\_OSpringBarleyPigs* |  |
    | *tov\_OTriticale* |  |
    | *tov\_OWinterBarley* |  |
    | *tov\_OWinterRape* |  |
    | *tov\_OWinterRye* |  |
    | *tov\_OWinterWheatUndersown* |  |
    | *tov\_PermanentGrassGrazed* |  |
    | *tov\_PermanentGrassTussocky* |  |
    | *tov\_PermanentSetaside* |  |
    | *tov\_Potatoes* |  |
    | *tov\_PotatoesIndustry* |  |
    | *tov\_SeedGrass1* |  |
    | *tov\_SeedGrass2* |  |
    | *tov\_Setaside* |  |
    | *tov\_SpringBarley* |  |
    | *tov\_SpringBarleyCloverGrass* |  |
    | *tov\_SpringBarleyGrass* |  |
    | *tov\_SpringBarleySeed* |  |
    | *tov\_SpringBarleySilage* |  |
    | *tov\_SpringRape* |  |
    | *tov\_SpringWheat* |  |
    | *tov\_Triticale* |  |
    | *tov\_WinterBarley* |  |
    | *tov\_WinterRape* |  |
    | *tov\_WinterRye* |  |
    | *tov\_WinterWheat* |  |
    | *tov\_WWheatPControl* |  |
    | *tov\_WWheatPToxicControl* |  |
    | *tov\_WWheatPTreatment* |  |
    | *tov\_AgroChemIndustryCereal* |  |
    | *tov\_WinterWheatShort* |  |
    | *tov\_Orchard* |  |
    | *tov\_OSBarleySilage* |  |
    | *tov\_OFieldPeasSilage* |  |
    | *tov\_FieldPeasStrigling* |  |
    | *tov\_MaizeStrigling* |  |
    | *tov\_SpringBarleyStrigling* |  |
    | *tov\_SpringBarleyCloverGrassStrigling* |  |
    | *tov\_WinterBarleyStrigling* |  |
    | *tov\_WinterRapeStrigling* |  |
    | *tov\_WinterRyeStrigling* |  |
    | *tov\_WinterWheatStrigling* |  |
    | *tov\_SpringBarleyPeaCloverGrassStrigling* |  |
    | *tov\_YoungForest* |  |
    | *tov\_SpringBarleyStriglingSingle* |  |
    | *tov\_SpringBarleyStriglingCulm* |  |
    | *tov\_WinterWheatStriglingSingle* |  |
    | *tov\_WinterWheatStriglingCulm* |  |
    | *tov\_MaizeSilage* |  |
    | *tov\_FodderGrass* |  |
    | *tov\_Lawn* |  |
    | *tov\_PermanentGrassLowYield* |  |
    | *tov\_SpringBarleyPTreatment* |  |
    | *tov\_OSpringBarleyExt* |  |
    | *tov\_OWinterWheatUndersownExt* |  |
    | *tov\_OWinterBarleyExt* |  |
    | *tov\_OMaizeSilage* |  |
    | *tov\_SpringBarleySKManagement* |  |
    | *tov\_Undefined* |  |

{

tov\_Carrots = 0,

tov\_CloverGrassGrazed1, // 1

tov\_CloverGrassGrazed2, // 2

tov\_FieldPeas, // 3

tov\_FodderBeet, // 4

tov\_Maize, // 5

tov\_NaturalGrass, // 6

tov\_NoGrowth, // 7

tov\_None, // 8

tov\_Oats, // 9

tov\_OBarleyPeaCloverGrass, // 10

tov\_OCarrots, tov\_OCloverGrassGrazed1, tov\_OCloverGrassGrazed2, tov\_OCloverGrassSilage1, tov\_OFieldPeas,

tov\_OFirstYearDanger, tov\_OGrazingPigs, tov\_OOats, tov\_OPermanentGrassGrazed, tov\_OPotatoes, // 20

tov\_OSeedGrass1, tov\_OSeedGrass2, tov\_OSetaside, tov\_OSpringBarley, tov\_OSpringBarleyClover, tov\_OSpringBarleyGrass,

tov\_OSpringBarleyPigs, tov\_OTriticale, tov\_OWinterBarley, tov\_OWinterRape, // 30

tov\_OWinterRye, tov\_OWinterWheatUndersown, tov\_PermanentGrassGrazed, tov\_PermanentGrassTussocky, tov\_PermanentSetaside,

tov\_Potatoes, tov\_PotatoesIndustry, tov\_SeedGrass1, tov\_SeedGrass2, tov\_Setaside, // 40

tov\_SpringBarley, tov\_SpringBarleyCloverGrass, tov\_SpringBarleyGrass, tov\_SpringBarleySeed, tov\_SpringBarleySilage,

tov\_SpringRape, tov\_SpringWheat, tov\_Triticale, tov\_WinterBarley, tov\_WinterRape, // 50

tov\_WinterRye, tov\_WinterWheat, tov\_WWheatPControl, tov\_WWheatPToxicControl, tov\_WWheatPTreatment, tov\_AgroChemIndustryCereal, //56

tov\_WinterWheatShort, tov\_Orchard, tov\_OSBarleySilage, tov\_OFieldPeasSilage, // 60

tov\_FieldPeasStrigling, tov\_MaizeStrigling, tov\_SpringBarleyStrigling, tov\_SpringBarleyCloverGrassStrigling,

tov\_WinterBarleyStrigling, tov\_WinterRapeStrigling, tov\_WinterRyeStrigling, tov\_WinterWheatStrigling, //68

tov\_SpringBarleyPeaCloverGrassStrigling, tov\_YoungForest,

tov\_SpringBarleyStriglingSingle, tov\_SpringBarleyStriglingCulm, tov\_WinterWheatStriglingSingle,

tov\_WinterWheatStriglingCulm, tov\_MaizeSilage, tov\_FodderGrass, tov\_Lawn, tov\_PermanentGrassLowYield,

tov\_SpringBarleyPTreatment,tov\_OSpringBarleyExt,tov\_OWinterWheatUndersownExt, tov\_OWinterBarleyExt, tov\_OMaizeSilage,

tov\_SpringBarleySKManagement,

tov\_Undefined // Must be in here and the last one as well!

}


- CJT
- MSVC
- ALMaSS Working Source
- Landscape
- tov\_declaration.h
- Generated on Thu Jan 10 2013 13:15:35 for ALMaSS Skylark ODdox by
   1.8.1.1
